# Leukotriene C_4_ biosynthesis in isolated August rat peritoneal leukocytes

**DOI:** 10.1155/S0962935196000610

**Published:** 1996-12

**Authors:** J. M. Huebner, R. R. Eversole, W. F. Jackson, C. D. Mackenzie, S. R. Stapleton, L. J. Beuving

**Affiliations:** 1 Department of Biological Sciences 5330 McCracken Hall Western Michigan University Kalamazoo MI 49008 USA; 2 Department of Pathology Michigan State University East Lansing MI 48824 USA

## Abstract

The mixed leukocyte population obtained from the peritoneum of the August rat is a potentially important experimental model of inherent eosinophilia that has not been well characterized. In the present study, isolated cell preparations generated a concentration-dependent release of leukotriene (LT) C_4_ when exposed to the Ca^2+^ ionophore A23187, reaching maximal stimulation at 5.0 μM. This response was inhibited by the 5-lipoxygenase activating protein antagonist MK-886 (0.1 μM), nominally Ca^2+^ and Mg^2+^-free incubation media and by activation of protein kinase C via phorbol 12-myristate 13-acetate (50 nM). These findings establish a model system for investigating LTC_4_ profiles contingent with innate peritoneal eosinophilia and are consistent with the hypothesis that cellular LTC_4_ biosynthesis is phosphoregulated.


Mediators of Inflammation, 5, 443–447 (1996)

### Leukotriene $C_4$ biosynthesis in isolated August rat peritoneal leukocytes

J. M. Huebner,[CA] R. R. Eversole,[1] W. F. Jackson,[1] C. D. Mackenzie,[2] S. R. Stapleton[1] and L. J. Beuving[1]

[1]Department of Biological Sciences, 5330 McCracken Hall, Western Michigan University, Kalamazoo, MI 49008, USA
[2]Department of Pathology, Michigan State University, East Lansing, MI 48824, USA

[CA]Corresponding Author
Tel: (616) 387 5631
Fax: (616) 387 2849
Email: huebner@wmich.edu

**THE mixed leukocyte population obtained from the peritoneum of the August rat is a potentially important experimental model of inherent eosinophilia that has not been well characterized. In the present study, isolated cell preparations generated a concentration-dependent release of leukotriene (LT) $C_4$ when exposed to the $Ca^{2+}$ ionophore A23187, reaching maximal stimulation at 5.0 μM. This response was inhibited by the 5-lipoxygenase activating protein antagonist MK-886 (0.1 μM), nominally $Ca^{2+}$ and $Mg^{2+}$-free incubation media and by activation of protein kinase C via phorbol 12-myristate 13-acetate (50 nM). These findings establish a model system for investigating $LTC_4$ profiles contingent with innate peritoneal eosinophilia and are consistent with the hypothesis that cellular $LTC_4$ biosynthesis is phosphoregulated.**

**Key words:** August rat, Eosinophilia, Leukotriene $C_4$, MK-886, Protein kinase C

### Introduction

The functional repertoire of both infiltrating and *in situ* inflammatory cells includes the biosynthesis and release of leukotrienes (LTs) derived from the 5-lipoxygenation of arachidonic acid (AA). Functionally, these compounds can be divided into two classes: (1) the dihydroxy acid $LTB_4$, which is a powerful chemotactic, aggregative and chemokinetic agent;[1] and (2) the cysteinyl-containing LTs $C_4$, $D_4$ and $E_4$; collectively termed the 'slow-reacting substance of anaphylaxis' or SRS-A.[2] Whereas $LTB_4$ has relatively few myotropic activities,[3] the C6 amino acid-substituted cysteinyl LTs are potent contractors of both vascular and non-vascular smooth muscle. The relative potencies of these compounds and their prominence in biological fluids and inflammatory exudates suggest a role in the pathophysiology of human bronchial asthma and other immediate hypersensitivity reactions.[4]

Under the appropriate conditions, it has now been shown that $Ca^{2+}$ mobilization can synergize with protein kinase C (PKC) activation to enhance the formation of LTs in human eosinophils,[5] human neutrophils[6–8] and murine macrophages.[9,10] This phenomenon can be demonstrated *in vitro* by the simultaneous application of the $Ca^{2+}$ ionophore A23187 and phorbol 12-myristate 13-acetate (PMA), a tumour-promoting phorbol ester which can activate PKC directly without initiating the hydrolysis of phosphatidylinositol.[11] It has been envisaged that the selective activities of these agonists may mimic the cellular events responsible for the release of free AA and/or the activation of 5-lipoxygenase (5-LO) as induced by physiologic stimuli.[7] In fact, there is evidence to suggest that the phosphorylation of specific target proteins by PKC may enhance the A23187-stimulated release of AA in certain cell types[12,13] as well as the biosynthetic activity of AA-selective phospholipase $A_2$ ($PLA_2$).[14,15]

In contrast, Kreiger *et al.*[16] have shown that PMA can effectively block the interleukin-3 (IL-3)-dependent formation of $LTC_4$ in human basophils, indicating that IL-3 signalling pathways and $LTC_4$ production in these cells do not require the activation of PKC. More recently, other laboratories have demonstrated that costimulation with PMA specifically attenuates $LTC_4$ production in differentiated human promyelocytic HL-60 cells challenged with ionophore or with saturating concentrations of exogenous $LTA_4$[17,18] the labile epoxide precursor to both $LTB_4$ and $LTC_4$.

We designed the present study to evaluate the

© 1996 Rapid Science Publishers capacity of August (AUG) rat mixed peritoneal leukocytes to generate $LTC_4$ when challenged with different concentrations of A23187 and to test the hypothesis that $LTC_4$ biosynthesis in this model is regulated via a PKC-specific phosphorylation mechanism. The AUG rat, which has a spontaneously high number of peritoneal eosinophils[19] that can be harvested by a relatively simple lavage procedure,[20] is a potentially important animal model of inherent eosinophilia that has not been well studied. Parasite-independent, non-induced examples of eosinophilia may prove particularly useful since both quantitative and qualitative differences exist between different preparations of eosinophils depending on the method of induction.[21]

### Materials and Methods

Except as noted, all reagents were purchased from Sigma Chemical Co. (St Louis, MO). Adult male AUG rats (Harlan/Olac, UK) aged 27–35 weeks were killed by $CO_2$ inhalation and the resident peritoneal cells harvested by washing the cavity with 50 ml of an ice-cold $CaCl_2$, $MgSO_4$ and $NaHCO_3$-free modified Hanks' Balanced Salt Solution (HBSS-1) buffered with 20 mM HEPES (pH 7.3). The lavage fluid was then aspirated by syringe and centrifuged at $200 \times g$ for 10 min at 4°C. Pelleted cells were washed, adjusted to yield $3.0 \times 10^6$ cells/ml with HBSS-1 and differential counts then determined from cytocentrifuge slides (Cytospin 3, Shandon Inc., Pittsburgh, PA) fixed and stained with a Diff-Quik Stain Set (Baxter Scientific Products, McGaw Park, IL). Cell suspensions obtained from two to three animals were pooled for all experiments.

Agonist and inhibitor stock solutions were prepared in dimethyl sulphoxide (DMSO), stored at −20°C and were serially diluted immediately before use with a HEPES-buffered HBSS supplemented with 2.4 mM $Ca^{2+}$ and 1.6 mM $Mg^{2+}$ at pH 7.3 (HBSS-2). The concentration of DMSO in the final reaction mixture was never greater than 0.4% (v/v). To elicit $LTC_4$ biosynthesis, $1.5 \times 10^6$ cells (500 μl) were incubated with different concentrations of A23187 in 500 μl of either HBSS-1 ($Ca^{2+}$ and $Mg^{2+}$-free) or HBSS-2 (final concentrations of 1.2 mM $Ca^{2+}$ and 0.8 mM $Mg^{2+}$) for 10 min (37°C, constant agitation). In other experiments, cells were stimulated with 0.5 μM A23187 in the presence of either 50 nM PMA or 0.1 μM MK-886, a potent 5-lipoxygenase activating protein (FLAP) inhibitor[22,23] (generously provided by Dr Anthony W. Ford-Hutchinson, Merck Frosst Centre for Therapeutic Research, Montreal, Canada). Viability cell counting was performed in some preparations after incubating 100 μl of the treated cell suspension for an additional 2 min at room temperature with a mixture of ethidium bromide (0.1 μg in 100 μl 0.1 M phosphate buffer) and fluorescein diacetate (2.5 μg in 100 μl 0.1 M phosphate buffer, diluted from a 5 mg/ml acetone stock solution). All other reactions were terminated by the addition of 3 ml ice-cold methanol and samples were stored at −70°C until assayed.

$LTC_4$ content was determined by acetylcholinesterase (AChE) enzyme immunoassay (EIA) (Cayman Chemical, Ann Arbor, MI). To prepare for analysis, samples were thawed and centrifuged at $1\,500 \times g$ for 10 min at 4°C to precipitate proteins. Supernatants were then evaporated to dryness in a heated (~50°C) water bath under a stream of nitrogen, reconstituted in 1 ml of assay buffer and stored overnight at 4°C. Each sample was assayed in duplicate according to the manufacturer's instructions. Results were interpolated from standard curves fit by third or fourth-order polynomial regression (Sigma Plot software, San Rafael, CA) of percentage displacement of bound $LTC_4$ AChE tracer versus $LTC_4$ concentration (pg/ml).

All data are presented as mean ± SE. Homogeneity of variance was confirmed by Hartley's test and a two-tailed, unpaired Student's $t$-test was used where appropriate.[24] A $p$ value $< 0.05$ was considered significant.

### Results

Cell preparations used in all experiments contained a mixture of macrophages (45 ± 1.0%), eosinophils (36 ± 0.8%), mast cells (11 ± 0.7%) and lymphocytes (8 ± 0.5%) (mean $= 38.0 \pm 2.0 \times 10^6$ total cells/harvest, range $= 29.5 \times 10^6$–$48.6 \times 10^6$, $n = 11$). Figure 1 (inset) shows the dose-dependent stimulation of cellular $LTC_4$ biosynthesis with 0.5–10 μM A23187. The maximum measured $LTC_4$ concentration (5.2 ± 0.3 ng $LTC_4/10^6$ cells; $n = 5$) was observed at 5.0 μM A23187, an increase nearly 65 times greater than the mean detected value observed in preliminary vehicle control experiments (56.0 ± 8.3 pg/$10^6$ cells; $n = 6$). Removal of both $CaCl_2$ and $MgSO_4$ from the incubation medium dramatically reduced the stimulatory effects of 0.5 μM A23187, reducing elicited $LTC_4$ levels from 577.3 ± 60.9 pg/$10^6$ cells ($n = 14$) to $< 30$ pg/$10^6$ cells ($n = 5$) (not shown). The FLAP antagonist MK-886[22,23] also inhibited A23187-induced LT synthesis by over 12-fold ($p < 0.05$, mean = 46.3 ± 3.0 pg/$10^6$

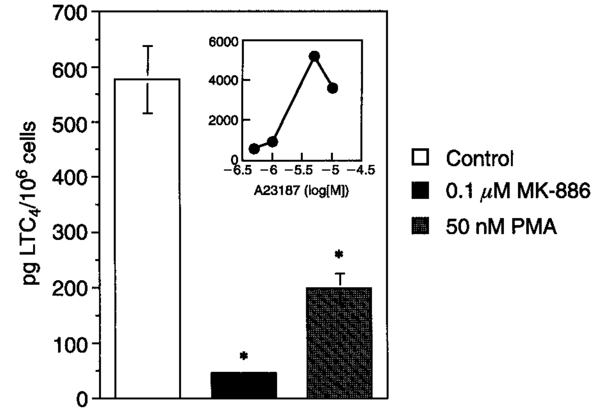

FIG. 1. A 5-lipoxygenase activating protein antagonist (MK-886) and PMA-mediated activation of protein kinase C attenuate 0.5 μM A23187-induced $LTC_4$ biosynthesis. Data are expressed as mean ± SE of at least six duplicate experiments. $^*p < 0.05$ compared with control value, as determined by the Student's *t*-test. Inset: Dose dependence of *in vitro* stimulation of $LTC_4$ biosynthesis by A23187. Data are expressed as the mean of at least four duplicate experiments and are shown ± SE.

cells, $n = 6$) (Fig. 1, column 1 *vs* column 2), indicating that the fundamental integrity of the biosynthetic pathway leading to the *de novo* formation of $LTC_4$ was retained in our isolates.

Next, we investigated the possible regulatory role of PKC in the synthesis of $LTC_4$ by co-incubating some isolates with 0.5 μM A23187 and 50 nM PMA, a myristoylated phorbol ester capable of directly activating PKC *in vitro*.[11] As evidenced in Fig. 1 (column 1 *vs* column 3), PMA reduced A23187-elicited $LTC_4$ release by nearly three-fold ($p < 0.05$, mean = 198.8 ± 26.1 pg/$10^6$ cells, $n = 7$), suggesting that cysteinyl leukotriene production in this model may be phosphoregulated. Because we found a slight, but significant difference in viability between the cells exposed to PMA ($p < 0.05$, mean = 83.5 ± 1.5% viable, $n = 5$) *vs* isolates treated with ionophore alone (mean = 90.7 ± 1.1% viable; $n = 6$), statistical comparisons between the two groups were also confirmed after adjusting the $LTC_4$ concentrations produced by PMA-treated cells by the mean percentage difference in viability between the two treatment groups (0.072). In this fashion, we sought to eliminate differences that could be attributable to cell death.

### Discussion

As part of a larger cooperative effort to understand the biology of the leukocyte population derived from the peritoneum of the syngeneic AUG rat strain, the present study demonstrates the concentration- and $Ca^{2+}/Mg^{2+}$-dependent stimulation of $LTC_4$ biosynthesis by the divalent cation ionophore A23187 and the inhibition of this response by MK-886,[23] a specific antagonist of FLAP activity.[22] Because MK-886 reportedly binds FLAP at a site corresponding to its putative AA binding domain,[25] thereby preventing the efficient transfer of AA to 5-LO, the findings presented here thus suggest crucial roles for $Ca^{2+}$ influx and substrate availability for the rate-limiting 5-LO enzyme.[26]

Previous studies have shown that PMA and suboptimal or threshold concentrations of A23187 synergistically potentiate the synthesis of $LTC_4$ in human eosinophils[5] and murine macrophages.[9,10] However, consistent with findings in human basophils[16] and differentiated substrains of the human promyelocytic HL-60 cell line,[17,18] our data suggest that PMA-mediated activation of PKC negatively regulates cysteinyl LT biosynthesis. Since it can also be demonstrated in other non-immunologic cell types that activation of PKC does not effectively modulate $LTC_4$ release in response to A23187,[26] it thus appears likely that the regulation of this biosynthetic pathway may differ substantially across species, compartments or cell phenotypes.

On the other hand, this interpretation does not fully explain the observations of other PMA-mediated effects, such as the potentiation of A23187-stimulated AA release,[12,13] the activation of AA-selective $PLA_2$[14,15] or the stimulation of prostanoid formation via the transcriptional activation of the cyclooxygenase-2 gene.[27,28] In fact, PKC is known to stimulate or enhance prostanoid biosynthesis in several cell types.[17,29–34] One plausible interpretation of these findings is the mechanism proposed by Ali *et al.*[17,35] whereby the phosphorylation of $LTC_4$ synthase, or a modulator of its activity, non-competitively inhibits $LTC_4$ biosynthesis, and the profile of eicosanoid mediators shifts from cysteinyl LTs towards the cyclooxygenase products of AA metabolism. The rapid accumulation of pharmacologically inactive $LTA_4$ would not necessarily be reflected in higher levels of $LTB_4$ production, since $LTA_4$ hydrolase is saturated at 40 μM.[17] If true, this hypothesis suggests a novel negative feedback mechanism whereby potent mediators such as $LTD_4$ can both attenuate their own production through binding receptors that trigger the activation of phosphatidylinositol-specific phospholipase C[36] and concomitantly stimulate the synthesis of other mediators (i.e. prostaglandins) some of which may counteract their physiological actions in tissues.[17]

Although it is unlikely that any one cell phenotype is directly responsible for all of the clinical manifestations accompanying the pathogenesis of inflammation,[37] it should be emphasized that secretory responses in our mixed cell population may not necessarily reflect the individual responses of the different cell types. Given the multitude of regulatory cascades that may be actively modulated by PKC,[38] as well as the remarkably cell-specific expression of 5-LO and $LTC_4$ synthase,[4] this complex issue requires further careful comparative study in order to identify the PKC protein substrates that affect the conversion of AA into various eicosanoids within each cellular context.

In conclusion, we now report that $Ca^{2+}$ ionophore A23187-induced $LTC_4$ synthesis by isolated AUG rat mixed peritoneal leukocytes is MK-886 sensitive and is both $Ca^{2+}/Mg^{2+}$ and concentration dependent. This suggests that these cells retain some of the functional characteristics exhibited by myeloid cells derived from other systems as well as the incipient signal transduction cascade leading to the *de novo* formation of $LTC_4$. Furthermore, we show that A23187-induced $LTC_4$ production is negatively regulated via PMA-mediated activation of PKC. Inasmuch as $LTC_4$ is the predominant bioactive 5-LO product of AA metabolism liberated by human eosinophils,[39] this model system of inherent eosinophilia could ultimately be exploited to furnish a basis for drug development targeted at lessening the severity of tissue reactions associated with inflammation.

### References

1. Ford-Hutchinson AW, Bray MA, Doig MV, Shipley ME, Smith MJ. Leukotriene $B_4$, a potent chemokinetic and aggregating substance released from polymorphonuclear leukocytes. *Nature* 1980; **286**: 264–265.
2. Samuelsson B. Leukotrienes: mediators of hypersensitivity reactions and inflammation. *Science* 1983; **220**: 568–575.
3. Piper PJ. Formation and actions of leukotrienes. *Physiol Rev* 1984; **64**: 744–761.
4. Lam BK, Austen KF. Leukotrienes: biosynthesis, release, and actions. In: Gallin JI, Goldstein IM, Snyderman R, eds. *Inflammation: Basic Principles and Clinical Correlates*, 2nd ed. New York: Raven Press, 1992; 139–147.
5. Tamura N, Agrawal DK, Townley RG. Leukotriene $C_4$ production from human eosinophils *in vitro*: role of eosinophil chemotactic factors on eosinophil activation. *J Immunol* 1988; **141**: 4291–4297.
6. McColl SR, Hurst NP, Cleland LG. Modulation by phorbol myristate acetate of arachidonic acid release and leukotriene synthesis by human polymorphonuclear leukocytes stimulated with A23187. *Biochem Biophys Res Commun* 1986; **141**: 399–404.
7. Liles WC, Meier KE, Henderson WR. Phorbol myristate acetate and the calcium ionophore A23187 synergistically induce release of LTB4 by human neutrophils: involvement of protein kinase C activation in regulation of the 5-lipoxygenase pathway. *J Immunol* 1987; **138**: 3396–3402.
8. McIntyre TM, Reinhold SL, Prescott SM, Zimmerman GA. Protein kinase C activity appears to be required for the synthesis of platelet-activating factor and leukotriene $B_4$ by human neutrophils. *J Biol Chem* 1987; **262**: 15370–15376.
9. Humes JL. Regulation of leukotriene formation in inflammatory cells. *Ann NY Acad Sci* 1988; **524**: 252–259.
10. Tripp CS, Mahoney M, Needleman P. Calcium ionophore enables soluble agonists to stimulate macrophage 5-lipoxygenase. *J Biol Chem* 1985; **260**: 5895–5898.
11. Castagna M, Takai Y, Kaibuchi K, Sano K, Kikkawa U, Naishizuka Y. Direct activation of calcium-activated, phospholipid-dependent protein kinase by tumor-promoting phorbol esters. *J Biol Chem* 1982; **257**: 7847–7851.
12. Halenda SP, Zavoico GB, Feinstein MB. Phorbol esters and oleoyl acetoyl glycerol enhance release of arachidonic acid in platelets stimulated by $Ca^{2+}$ ionophore A23187. *J Biol Chem* 1985; **260**: 12484–12491.
13. Peters-Golden M, McNish RW, Sporn PH, Balazovich K. Basal activation of protein kinase C in rat alveolar macrophages: implications for arachidonate metabolism. *Am J Physiol* 1991; **261**: L462–L471.
14. Lin L-L, Lin AY, Knopf JL. Cytosolic phospholipase $A_2$ is coupled to hormonally regulated release of arachidonic acid. *Proc Natl Acad Sci USA* 1992; **89**: 6147–6151.
15. Lin L-L, Wartmann M, Lin AY, Knopf JL, Seth A, Davis RJ. $cPLA_2$ is phosphorylated and activated by MAP kinase. *Cell* 1993; **72**: 269–278.
16. Krieger M, von Tscharner V, Dahinden CA. Signal transduction for interleukin-3-dependent leukotriene synthesis in normal human basophils: opposing role of tyrosine kinase and protein kinase C. *Eur J Immonol* 1992; **22**: 2907–2913.
17. Ali A, Ford-Hutchinson AW, Nicholson DW. Activation of protein kinase C down-regulates leukotriene $C_4$ synthase activity and attenuates cysteinyl leukotriene production in an eosinophilic substrain of HL-60 cells. *J Immunol* 1994; **153**: 776–788.
18. Kargman S, Ali A, Vaillancourt JP, Evans JF, Nicholson DW. Protein kinase C-dependent regulation of sulfidopeptide leukotriene biosynthesis and leukotriene $C_4$ synthase in neutrophilic HL-60 cells. *Mol Pharmacol* 1994; **45**: 1043–1049.
19. Spry CJF. *Eosinophils*. New York: Oxford University Press, 1988; 123–127.
20. Mackenzie CD, Jungery M, Taylor PM, Ogilvie BM. The in-vitro interaction of eosinophils, neutrophils, macrophages and mast cells with nematode surfaces in the presence of complement or antibodies. *J Path* 1981; **133**: 161–175.
21. Cook RM, Musgrove NRJ, Ashworth RF. Activity of rat peritoneal eosinophils following induction by different methods. *Int Archs Allergy Appl Immun* 1987; **83**: 423–427.
22. Rouzer CA, Ford-Hutchinson AW, Morton HE, Gillard JW. MK886, a potent and specific leukotriene biosynthesis inhibitor blocks and reverses the membrane association of 5-lipoxygenase in ionophore-challenged leukocytes. *J Biol Chem* 1990; **265**: 1436–1442.
23. Gillard J, Ford-Hutchinson AW, Chan C, *et al.* L-663,5396 (MK-886) (3-[1-(4-chlorobenzyl)-3-t-butyl-thio-5-isopropylindol-2-yl]-2,2-dimethylpropanoic acid), a novel, orally active leukotriene biosynthesis inhibitor. *Can J Physiol Pharmacol* 1989; **67**: 456–464.
24. Ott L. *An Introduction to Statistical Methods and Data Analysis*, 3rd ed. Boston: PWS-Kent, 1988; 415–416.
25. Ford-Hutchinson AW. Leukotriene C4 synthase and 5-lipoxygenase activating protein: regulators of the biosynthesis of sulfido-leukotrienes. *Ann NY Acad Sci* 1994; **744**: 78–83.
26. Huber W, Trautmann M, Becker I, Schenck U, Peskar BM, Schepp W. Leukotriene $B_4$ and $C_4$ production in isolated gastric mucosal cells. *Am J Physiol* 1993; **265**: G1021–G1028.
27. Gilbert RS, Herschman HR. Macrophage nitric oxide synthase is a glucocorticoid-inhibitable primary response gene in 3T3 cells. *J Cell Physiol* 1993; **157**: 128–132.
28. Kujubu DA, Fletcher BS, Varnum BC, Lim RW, Herschman HR. TIS10, a phorbol ester tumor promoter-inducible mRNA from Swiss 3T3 cells, encodes a novel prostaglandin synthase/cyclooxygenase homologue. *J Biol Chem* 1991; **266**: 12866–12872.
29. Humes JL, Sadowski S, Galavage M, *et al.* Evidence for two sources of arachidonic acid for oxidative metabolism by mouse peritoneal macrophages. *J Biol Chem* 1982; **257**: 1591–1594.
30. Ota S, Hata Y, Terano A, *et al.* Roles of $Ca^{2+}$ and protein kinase C in regulation of prostaglandin $E_2$ release by cultured rabbit gastric epithelial cells. *Dig Dis Sci* 1993; **38**: 1426–1434.
31. Peters-Golden M, Coburn K, Chauncey JB. Protein kinase C activation modulates arachidonic acid metabolism in cultured alveolar epithelial cells. *Exp Lung Res* 1992; 18: 535–551.
32. Simonson MS, Wolfe JA, Konieczkowski M, Sedor JR, Dunn MJ. Regulation of prostaglandin endoperoxide synthase gene expression in cultured rat mesangial cells: induction by serum via a protein kinase-C-dependent mechanism. *Mol Endocrinol* 1991; **5**: 441–451.
33. Thore CR, Nam M, Busija D. Phorbol ester-induced prostaglandin production in piglet cortical astroglia. *Am J Physiol* 1994; **267**: R34–R37.
34. Yokota K. Cellular mechanism of synergistic stimulation of $PGE_2$ production by phorbol diester and $Ca^{2+}$ ionophore A23187 in cultured madin-darby canine kidney cells. *Arch Biochem Biophys* 1991; **288**: 192–201.
35. Ali A, Nicholson DW, Ford-Hutchinson AW. Characterization of human $LTC_4$ synthase. *Adv Prostaglandin Thromboxane Leukotriene Res* 1995; **23**: 171–173.

36. Crooke ST, Mong S, Sarau HM, Winkler JD, Vegesna VK. Mechanisms of regulation of receptors and signal transduction pathways for the peptidyl leukotrienes. *Ann NY Acad Sci* 1988; **524**: 153–161.
37. Kay AB. Asthma and inflammation. *J Allergy Clin Immunol* 1991; **87**: 893–910.
38. Holz RW, Bittner MA. The role of protein kinase C in exocytosis. In: Kuo JF, ed. *Protein Kinase C*. New York: Oxford University Press, 1992; 269–289.
39. Weller PF, Lee CW, Foster DW, Corey EJ, Austen KF, Lewis RJ. Generation and metabolism of 5-lipoxygenase pathway leukotrienes by human eosinophils: predominant production of leukotriene $C_4$. *Proc Natl Acad Sci USA* 1983; **80**: 7626–7630.

ACKNOWLEDGEMENTS. This work was supported by grants to J.M.H. from the Graduate Student Research Fund, Western Michigan University and conducted in part during the tenure of a fellowship to J.M.H. from the Center for Research in Environmental Signal Transduction (CREST) at this institution. W.F.J. was supported by PHS grant HL 32469. The authors gratefully acknowledge Dr Frank F. Sun and Bruce M. Taylor of Pharmacia & Upjohn, Inc. for their assistance with the EIA.

**Received 14 August 1996;**
**accepted in revised form 3 September 1996**
